# Asymmetry, division of labour and the evolution of ageing in multicellular organisms

**DOI:** 10.1098/rstb.2019.0729

**Published:** 2021-04-26

**Authors:** Ido Pen, Thomas Flatt

**Affiliations:** ^1^ Theoretical Research in Evolutionary Life Sciences, Groningen Institute for Evolutionary Life Sciences, University of Groningen, 9747 AG, Groningen, The Netherlands; ^2^ Department of Biology, University of Fribourg, Chemin du Musée 10, CH-1700 Fribourg, Switzerland

**Keywords:** ageing, asymmetry, division of labour, germ-line, soma, multicellularity

## Abstract

Between the 1930s and 1960s, evolutionary geneticists worked out the basic principles of why organisms age. Despite much progress in the evolutionary biology of ageing since that time, however, many puzzles remain. The perhaps most fundamental of these is the question of which organisms should exhibit senescence and which should not (or which should age rapidly and which should not). The evolutionary origin of ageing from a non-senescent state has been conceptually framed, for example, in terms of the separation between germ-line and soma, the distinction between parents and their offspring, and—in unicellular organisms—the unequal distribution of cellular damage at cell division. These ideas seem to be closely related to the concept of ‘division of labour' between reproduction and somatic maintenance. Here, we review these concepts and develop a toy model to explore the importance of such asymmetries for the evolution of senescence. We apply our model to the simplest case of a multicellular system: an organism consisting of two totipotent cells. Notably, we find that in organisms which reproduce symmetrically and partition damage equally, senescence is still able to evolve, contrary to previous claims. Our results might have some bearing on understanding the origin of the germ-line–soma separation and the evolution of senescence in multicellular organisms and in colonial species consisting of multiple types of individuals, such as, for example, eusocial insects with their different castes.

This article is part of the theme issue ‘Ageing and sociality: why, when and how does sociality change ageing patterns?'

## 1. Introduction

The basic population genetic principles whereby ageing (senescence) evolves were understood by the 1960s, based on ground-breaking insights by Peter B. Medawar [1,2], George C. Williams [3] and especially William D. Hamilton [4]: because the strength of selection declines with age, ageing evolves owing to the spreading of alleles that have deleterious effects late in life but that have either neutral or beneficial effects on fitness early in life (‘mutation accumulation’ and ‘antagonistic pleiotropy' theories of the evolution of ageing; reviewed in [5–8]). Yet, given the remarkable diversity of patterns of ageing and lifespan among different taxa [9–11] and the apparent existence of organisms that age slowly or not at all [12–15], an even more fundamental question curiously remains poorly understood: which organisms should age in the first place and which should not [3,8,11,16–19]?

Beginning with August Weismann [20–22], various criteria aimed to demarcate ageing versus non-ageing organisms have been put forward, including for instance the separation between germ-line and soma, an asymmetry between parents and their offspring, and the asymmetrical distribution of somatic damage at cell division in unicellular organisms [3,5,17,18,23–28]. However, empirical or theoretical studies of these phenomenological ‘demarcation' criteria have so far rarely been undertaken (for some exceptions see e.g. [19,24,26,27,29]).

Here, we first provide a review of the role of the above-mentioned ‘asymmetries' in the evolution of senescence and argue, in a similar vein to Kirkwood [25], that they are closely related to the fundamental issue of ‘division of labour' (and hence trade-offs) between reproductive and somatic functions, a point first made in the context of senescence by Weismann [21]. Second, we develop a toy model of division of labour and ageing for the simplest kind of multicellular organism: an organism consisting of only two, initially completely identical, totipotent cells. Using this deliberately oversimplified model, and making minimal assumptions, we examine the conditions whereby such a system evolves division of labour between reproductive and somatic functions and how this might affect senescence.

The aim of our review and simulations is to offer some tentative conjectures that might be relevant for understanding the evolution of germ-line–soma sequestration and the evolution of ageing in multicellular and colonial species, for example in eusocial insects where the reproductive division of labour is often correlated with major differences in lifespan between castes.

## 2. Which organisms should age and which should not?

The question of which organisms should age and which should not, or at least do so only very slowly, is a long-standing but still poorly understood problem in the biology of ageing [8,11,16]. Is ageing truly universal among organisms and thus inevitable [18,24], or are there species that can genuinely defy senescence [8,14,15]? What are the fundamental differences between organisms that age and those that do not? Pondering this major puzzle, various authors have suggested criteria that might be used to delineate ageing versus non-ageing organisms [3,5–7,18,20,23,24].

Most notably, Weismann [20–22] (and subsequently also Williams [3]) claimed that ageing should occur only in those organisms that exhibit a clear separation between a potentially immortal germ-line and a mortal soma whose function is to support reproduction at the expense of its own maintenance and survival (also see discussion in [5,8,17,18,23,28,29]), an idea that forms the cornerstone of Kirkwood's ‘disposable soma' theory of the evolution of ageing [28,30–32] and that is consistent with the key role of trade-offs in shaping the evolution of ageing [3,5–8,17]. Under this view, ageing might be seen as a phenomenon of the soma [3] and should be prevalent among metazoans with a clear delineation of germ-line and soma (so-called Weismannian organisms).

However, as pointed out by Partridge & Barton [23], even a hypothetical organism consisting only of germ cells or gametes (and for which therefore no distinction between germ-line and soma exists) would have a non-zero probability of death or of becoming unable to produce gametes; consequently, the strength of selection on survival and reproduction would decline with age, and ageing would be expected to evolve. Indeed, in some sense, at an evolutionary time scale, even the germ-line can be said to age [17,23]: without sexual reproduction, clonal lineages accumulate deleterious mutations (Muller's ratchet), whereas sexual reproduction and recombination allow the germ-line to rid itself of DNA damage. From this perspective, the evolution of sex seems to be essential for preventing the germ-line from undergoing accumulation of age-dependent deleterious mutations and thus senescence [17,23,33]. Importantly, however, some asexual metazoans that lack clear germ-line sequestration can apparently undergo senescence [26], thus ruling out the universality of the germ-line–soma criterion for the evolution of ageing. In support of these observations, an analysis of 308 multicellular taxa suggests that ageing in organisms without germ-line–soma separation is widespread, while some species that do have clear germ-line–soma differentiation do not appear to undergo senescence [19].

As argued by Stearns [18], implicit in Weismann's claim is the assumption that in organisms with germ-line–soma separation reproduction is asymmetrical (i.e. the germ-line specializes in reproduction and is potentially immortal, but the soma ages and dies), whereas organisms (such as single-celled protozoans) that reproduce by fission have symmetrical reproduction. In a similar vein, Williams [3, p. 403] remarked that ‘Fissile animals would appear at first sight to lack a soma, but often the products of fission are not altogether similar. It may be physiologically justified to regard one as parent and the other as offspring. The asexual reproduction of turbellarian flatworms, for example, is often termed fission, but the division is transverse and separates a head end from a quite different tail end'. Hence, following Williams' reasoning [3], and as also discussed by Charlesworth [6] and Bell [29], truly symmetrically reproducing organisms (e.g. fissile species that divide into equal parts, symmetrically dividing unicellular organisms) should not age because they lack a clear distinction between parent and offspring.

This point was made explicit by Partridge & Barton [23, p. 310], who stated that ‘The critical requirement for the evolution of ageing is that there be a distinction between a parent individual and the smaller offspring for which it provides. If the organism breeds by dividing equally into identical offspring, then the distinction between parent and offspring disappears, the intensity of selection on survival and reproduction will remain constant and individual ageing is not expected to evolve.' (also see [5,17,24,34,35]). Thus, according to these authors, the key criterion for the evolution of ageing is asymmetrical reproduction or, in other words, parent–offspring asymmetry [6,17,18,23,24,29]. From this point of view, without a clear delineation between parents and offspring, there would be no real biological age structure (i.e. in some sense, parent and offspring would have identical biological age, even if they have different chronological age), and the force of selection would not decline with age. A mere asymmetry between parent and offspring is, however, not sufficient: the parent must be *older* than the offspring. Moreover, this age difference must translate into age-dependent physiological effects that impact fitness. Accumulated damage, for example, might represent both a physiological correlate of age (a 'biomarker' of age) as well as a physical manifestation of the senescence process. Reproduction can therefore be seen as being ‘rejuvenating' in the sense that the parent retains aged structures, while in the offspring the ‘biological clock' is reset (see [24] and discussion and references therein).

This criterion led to the experimental prediction that asymmetrically dividing bacteria or other unicellular organisms should, at least under some conditions, exhibit senescence. Experiments by Ackermann *et al*. [36] with the asymmetrically reproducing bacterium *Caulobacter crescentus* clearly confirmed this expectation. Subsequent work by Stewart *et al.* [37] found that senescence also occurs in *Escherichia coli*, a bacterium that divides symmetrically into seemingly identical (!) daughter cells but which exhibits a form of subcellular asymmetry where molecular structures are distributed unequally among daughter cells, i.e. daughter cells either retain an ‘old' cell pole (from a previous division) or they receive a new (young) cell pole created during division. Cells with older poles accumulate protein aggregates, and this accumulation is associated with a greater than 30% loss of reproductive ability (i.e. ageing) of the ‘old pole' cell lineage as compared with cells that have inherited a new, ‘young' pole at cell division [38]. Further work in this system has shown that, when damage accumulation exceeds a certain threshold, the ageing lineage becomes mortal, whereas the rejuvenated lineage within the same population of cells remains immortal [39]. For further discussion and some additional examples of ageing in unicellular organisms see for instance [24,25,40–42].

Building on these findings, Ackermann *et al.* [24] developed a simple model of ageing in unicellular organisms. Remarkably, they found that, starting from completely symmetrical initial conditions, selection favours the asymmetrical distribution of cellular damage and leads to the evolution of an ageing parent that retains the damage and a rejuvenated daughter cell that is initially damage-free. Thus, in this model, parent–offspring asymmetry—and hence ageing, according to the criterion outlined by Partridge & Barton [23]—emerge as a direct consequence of asymmetric damage distribution and imperfect repair. Complementary theoretical models of damage partitioning in unicellular organisms, as well as empirical data from fission yeast (*Schizosaccharomyces pombe*), have reached similar conclusions [43–46]. In further support of the notion that parent–offspring asymmetry plays a key role in the evolution of senescence, the asexually reproducing (fissile) marine oligochaete *Paranais litoralis* lacks clear germ-line sequestration, undergoes senescence, and parents produce offspring by ‘regular growth posterior to a fission zone located at a constant number of segments from the parental head. The offspring contains no segment of the parent and is composed entirely of new tissue.' [26, p. 9921].

Together, the body of work discussed above seems to rule out the basic idea that the separation of germ-line and soma is a prerequisite for the evolution of senescence and suggests that the central requirement for the origin of ageing might be the asymmetry between parents and offspring [26]. One can, however, not easily fail to notice some potentially profound similarities between the different concepts discussed above, as we discuss next.

## 3. Division of labour and the evolution of senescence

Let us now consider how it happened that the multicellular animals and plants, which arose from unicellular forms of life came to lose this power of living forever. The answer to this question is closely bound up with the principle of division of labour … the first multicellular organism was probably a cluster of similar cells, but these units soon lost their original homogeneity … the single group would come to be divided into two groups of cells, which may be called somatic or reproductive. [21, p. 28]

We believe that the evolution of somatic differentiation …, and not germ-line sequestration, was the necessary condition for the evolution of senescence.

[26, p. 9922]

In an editorial entitled ‘Asymmetry and the origins of ageing’, Kirkwood [25] posited that the criteria outlined above can all be viewed, at an abstract level, as representing different manifestations of the same sort of fundamental asymmetry (or ‘differentiation') which results from the principle of division of labour [47–50], an idea already considered by Weismann [21] (also see [42,50]). The appeal of this idea is that it can potentially provide a rather natural explanation for the different asymmetries that might be involved in the evolutionary origin of senescence, from asymmetrical damage distribution in bacteria to germ-line–soma differentiation seen in numerous metazoans. This unifying concept might thus be seen as a generalization of the parent–offspring asymmetry criterion discussed by Williams [3], Charlesworth [6], Bell [29], Partridge & Barton [23] and others. From this vantage point, the separation of germ-line and soma might be a sufficient example of such a generalized asymmetry, but it might not be a necessary one [42].

Following Kirkwood, the origin of ageing might be intimately linked to the division of labour among (or differentiation of) reproductive versus somatic functions that support survival (including e.g. maintenance, repair, damage sequestration, foraging, resource acquisition, growth), which together determine the fitness (i.e. the expected contribution to the future gene pool) of an organism or an organismal ‘system' (e.g. a colony of closely related individuals). There is typically some ‘tension' between these energetically or otherwise costly functions; the ‘severity' of this ‘tension' depends on the details of the trade-offs that exist between them. In unicellular organisms, for example, these fitness-related functions coexist within a single cell; in non-colonial metazoans, they coexist among different tissues or compartments within a single individual; and in colonial species these functions are fulfilled by different types of individuals within a colony. If trade-offs between these functions are sufficiently acute, functional specialization or differentiation (i.e. division of labour) can ‘ease' this ‘tension' by temporally and/or spatially separating them among the constituent parts of the system.

Let us consider some examples in support of this idea. As mentioned above, in unicellular organisms damage can be asymmetrically (spatio-temporally) distributed among cells when they divide, with one cell retaining the damage and the other being born undamaged [24,37], thus leading to a germ-line-like and a soma-like lineage of cells [50]. Notably, this sort of parent–offspring asymmetry does not require any external, phenotypically visible asymmetry [24,37], for example, in terms of offspring being smaller than the parent [23]. Similarly, in many metazoans that exhibit ageing, germ-line sequestration separates reproductive processes from somatic maintenance functions [17,27,31,51,52], whereas in others that age but lack clear germ-line sequestration, division of labour between reproductive and somatic functions can nonetheless be observed. In the asexual oligochaete *P. litoralis*, for instance, germ cells develop from undifferentiated pluripotent stem cells while differentiated somatic cells seem to be stably committed to their somatic functions [26]. According to these authors, the stability of terminal somatic differentiation (not germ-line sequestration) prevents somatic cells from becoming germ cells and renders them disposable [26, p. 9922]: during the evolution of multicellularity, ‘Some cells lost their capacity to produce a new individual; they became committed to functions other than reproduction. Free from the need for immortality, the metazoan soma became liable to undergo senescence'. The perhaps most famous example of such a division of labour is found in eusocial insects, where some colony members specialize in reproduction (queens in eusocial hymenopterans and additionally kings in termites) and others specialize in somatic tasks including, for example, foraging, colony hygiene, nest defence and social immunity (e.g. see [47,53] and references therein).

A major consequence of such a division of labour is that the constituent parts of multicellular (or colonial) organisms or the cellular lineages of unicellular organisms which fulfil these separable functions exhibit different mortality trajectories and hence lifespans (e.g. because of inherent differences in baseline mortality and/or in age-dependent mortality, for example, owing to different rates of damage accumulation or investment into repair) (also see [3,50]). In bacteria, asymmetrical damage distribution at cell division leads to the emergence of a damage-accumulating, ageing parent and a rejuvenated offspring cell whose biological clock is reset [24,50]; in Weismannian organisms that reproduce sexually, the potentially immortal germ-line is frequently being repaired and freed of damage, whereas the sterile, disposable soma performs essential maintenance tasks, accumulates damage at a faster rate and is mortal [17,27,31,52]; in eusocial insects, the long-lived reproductive queens and kings [54] might be viewed as being conceptually similar to the germ-line, while the short-lived workers are akin to the soma [55–59]. Such systems (or lineages) are thus characterized by relatively long-lived and relatively short-lived, disposable constituents; the longevity of one part of the system seems to come at the price of the disposability of another part [25,42,50]. This idea might thus also be relevant for our understanding of varying rates of functional senescence of different cell types, tissues, organs or body parts within an individual organism: although Williams had claimed that, because 'natural selection will always be in greatest opposition to the decline of the most senescence-prone system', ageing in a multi-component system should be synchronized [3, p. 406] (also see [60]), empirical evidence suggests contrarywise that many systems exhibit 'mosaic ageing', i.e. differential senescence of different constituent parts [61–65]. Moreover, it is also clear that the division of labour among reproductive versus somatic functions might not be absolute or perfect: for example, the ‘barrier' between germ-line and soma (the so-called ‘Weismann barrier') is known to be leaky, and the germ-line itself can manifestly age [66]—the main point, however, is that the germ-line ages less rapidly than the soma.

Indeed, several lines of evidence are consistent with the view that, with the division of labour, different reproductive and somatic functions are characterized by different lifespans. For example, Goldsby *et al*. [27], using a computational model of ‘digital' multicellular organisms, found that such organisms can evolve differentiation into reproductive versus somatic functions and that somatic cells age faster. Intriguingly, recent work on replicative senescence in yeast (*Saccharomyces cerevisiae*) has found that wild-type cells can exhibit two distinct, apparently mutually exclusive modes of ageing: mode 1 cells are characterized by increased instability of nucleoli, whereas mode 2 cells show a decline of mitochondrial function, with mode 2 cells being shorter lived and having a greater cell cycle length than mode 1 cells [67]. Similarly, empirical data from eusocial insects suggest that the degree of division of labour (i.e. the extent of caste differentiation) is positively correlated with the difference in lifespan between long-lived reproductive individuals (queens, kings) and short-lived non-reproductive individuals (workers); this difference in lifespan between the castes tends to be smaller in species with totipotent workers which have the potential to develop into reproductive individuals [68–72]. Division of labour in terms of damage sequestration might also lead to different lifespans of distinct parts or modules in modular organisms: individuals might limit damage at the organismal level by shedding such damaged modules and parts. The ‘disposability' of such damaged parts or modules at the lower level of organization might help to promote the longer life of the integrated modular organism as a whole. This hypothesis, originally proposed by Finch [9], has recently received empirical support from a comparative analysis of senescence in plants [73]; the role of modularity in affecting ageing in animals, however, remains less clear.

Research on volvocalean green algae (some of which represent unicellular organisms, while others can form multicellular colonies) can serve to illustrate some of these points [74–79]. Unicellular forms of these green algae are subject to a structural trade-off between flagellar locomotion and reproduction (e.g. [72–75]): in these small planktonic algae, survival depends on motility (e.g. important for nutrient acquisition, avoiding sinking to the bottom of the water column, escaping predators, etc.), yet motile cells cannot divide while dividing cells cannot move because both processes (motility, mitotic cell division) use the same organelles either as basal bodies or as centrioles (also see [47–49,80]). This fundamental ‘tension' or trade-off between survival and reproductive functions has been solved by colonial forms of these green algae via division of labour: they have evolved sterile cells that are specialized in motility, while other cells have become specialized in reproduction—a situation that is similar to the differentiation of germ-line and soma [77–79]. Theoretical work by Michod [77] suggests that if the trade-off curve relating survival and reproduction is convex, colony formation and division of labour should be favoured, whereas this should not happen if the trade-off function is concave (also see [81,82]). A convex trade-off means that at one end of the trade-off curve, a decrease in, say, survival is compensated for by more than a proportional increase in fecundity, thus favouring specialization in fecundity, whereas at the other end of the curve, a decrease in fecundity is compensated for by more than a proportional increase in survival, thus favouring specialization in survival. By contrast, a concave function implies that there are diminishing returns on investment into either component, so that specialization into, for example, reproductive versus somatic functions would not pay off [77].

If the division of labour is favoured, it harbours the potential for conflict and competition—e.g. over contributing to the next generation—among the specialized modules, parts or functions; conflict or competition can, however, to some extent be overcome if, for example, the specialized modules or parts are genetically closely related [47,48]. In clonally reproducing unicellular organisms, for instance, the ageing mother and the rejuvenated offspring clearly represent distinct ‘individual’ cells but, from the point of view of selection, they share the same genotype and can thus be thought of as representing a single ‘individual’ [3]. Similarly, in many multicellular organisms, the constituent cells are typically genetically identical, at least to the first order of approximation (i.e. with the exception of somatic mutations). In general, for such multi-level or multi-component biological systems, fitness optimization requires that selection at the higher level (e.g. the multicellular individual, the colony) supersedes selection at the lower level (e.g. the individual components of the integrated system) and that conflict or competition at the lower level be minimized [47,48]. Yet, using a model of intra-organismal somatic selection, Nelson & Masel [83] have recently suggested that such conflict suppression might be imperfect and that intercellular somatic competition might make ageing inevitable in multicellular organisms. (If correct, this would obviously not rule out that some organisms might age extremely slowly, maybe immeasurably so.)

The above insights into how trade-offs affect the division of labour have been generalized by Rueffler *et al*. [49] in a theoretical analysis of the costs and benefits of functional specialization in terms of accelerating and decelerating functions that map the phenotypes of different functional modules to performance. Similar to the above arguments, this work shows that the division of labour is favoured when the gain in performance in one function due to specialization of a module outweighs the loss of performance in the same task due to specialization of other modules on other functions. Interestingly, the findings by Rueffler *et al.* [49] also suggest that, if functional modules are frequently lost or damaged, selection for ‘robustness' (i.e. the reliable ability to perform both tasks) might disfavour the evolution of the division of labour. For example, performance in one task might affect survival, whereas performance in another task might affect reproduction. When survival and reproduction represent multiplicative fitness components, and if either one of them is strongly decreased, then overall fitness will be strongly decreased too [49].

It is noteworthy that the process of ageing typically represents exactly such a loss of fitness, i.e. a decrease of reproduction and survival with increasing age. How then can the evolution of ageing and division of labour be reconciled? The key to this problem might be that for organisms for which the force of selection declines more rapidly with age than for other organisms for which this decline is markedly weaker, division of labour between reproductive and somatic functions might nonetheless pay off and be selectively favoured, especially under high extrinsic mortality. As Kirkwood has put it: ‘There is therefore little advantage to be gained from investment in potential somatic immortality when in practice the return on investment may not be realised. Taking account of the level of environmental mortality, the better course will always be to reduce the investment into somatic repair and maintenance to a level which ensures only that the soma remains in good condition through its normal expectation of life in its natural environment and to use extra resources liberated by this action to increase reproduction.' [31]. (N.B. This argument assumes that damage accumulation is the predominant cause of senescence; for an alternative view see [84].)

We find it telling in this context that many organisms that seem to age very slowly, if at all, including for instance various basal metazoans (such as *Hydra*) and plants [14,85], possess pluri- or totipotent stem cells that can differentiate into every cell type of the body, thereby endowing these organisms with a remarkable capacity for regeneration and rejuvenation [86–88] (also see discussion in [3,11]). It is intriguing that such pluri- or totipotency at the cellular level seems to be, at least to some extent, *antithetical* to the division of labour among reproductive and somatic functions discussed above. On the other hand, perennial plants also seem to be able to remove accumulated damage through the death of disposable soma-like cells or structures [88], as already alluded to above [73]. If so, this would again be consistent with the notion that division of labour (i.e. between pluripotent stem cells with germ-line-like features and cells or tissues with some soma-like properties) plays a major role in the evolution of ageing [26], perhaps even for cases of negligible senescence. Remarkably, recent work by Aanen & Debets [89] and others suggests that the distinction between germ-line and soma might be more profound than previously thought and might extend to unicellular organisms: for instance, based on empirical observations that at high cell density mutation rates are strongly reduced in both prokaryotic and eukaryotic unicellular organisms, and that some ‘mother' cells continue dividing whereas their ‘offspring' cells stop dividing, Aanen and colleagues have found evidence to suggest that the observed reduction in mutation rate at high density can be explained if mother cells preferentially retain the template DNA strands, thereby confining new mutations to non-dividing daughter cells—a phenomenon similar to the separation of germ-line and soma. This so-called ‘Immortal Strand Hypothesis' [90] is also consistent with data on mutation accumulation from fungi [91–94].

In summary, our review of the literature above suggests that various manifestations of the principle of division of labour might be central to our understanding of the evolution of senescence. We therefore speculate that the rate (speed) of senescence observed among different organisms (or their constituent ‘parts'), from slow to rapid ageing, might depend critically on the details of both the extent and the stability of division of labour or differentiation (and hence trade-offs) between various somatic (e.g. damage sequestration, repair) and reproductive functions.

To further explore these issues, we now turn to discussing a simple model for the evolution of division of labour and ageing in multicellular organisms. Previous models of division of labour have investigated the evolution of germ-line–soma differentiation and multicellularity [77,81,95] but not directly in the context of ageing, with a few exceptions: using an evolutionary model, Radzvilavicius *et al.* [96] have suggested that selection for mitochondrial quality has driven the evolution of the germ-line, with germ-line sequestration reducing mutational input; in a similar vein, Goldsby *et al.* [27] used a computational model of evolvable ‘digital' multicellular organisms to show that such organisms can evolve division of labour between reproductive and somatic (‘dirty work', i.e. somatic maintenance) functions, with somatic cells undergoing more rapid senescence, while leaving the reproductive cells ‘protected'. Our starting point here is the simplest imaginable case of a multicellular system: an organism or ‘colony' that consists of two initially totipotent cells. We employ this toy model to begin to probe into the potential roles of different kinds of asymmetries in the evolution of division of labour and their consequences for ageing.

## 4. The model

While pondering how to design a minimalistic model, we formulated two basic *desiderata* that the model must fulfil:

(1) Ageing—that is, an increase of mortality and/or decrease of fecundity with advancing age—or its absence must be able to evolve.
(2) Separation of germ-line and soma must be possible, but also its absence. Hence, individuals must have at least two cell types, one of which can in principle monopolize the production of gametes or clones.

Thus, we embarked on building an individual-based simulation model of a population containing *N* bicellular adult organisms. The life cycle starts with a single cell, which doubles once during development, giving rise to an adult organism that consists of two cells, the mother cell designated ‘type 1' and the daughter cell ‘type 2'. The cells 'know' their type because we want them to (eventually) be able to condition their behaviour on their type, although at the start of each simulation the two cells act identically (i.e. symmetrically). After development, both cells are endowed with the same amount of resources, which they can split, according to genetically determined type-specific reaction norms, among three energetically costly processes: foraging for resources, repair of cellular damage, and reproduction; thus, we assume the existence of trade-offs between reproduction and somatic maintenance functions, for example, consistent with William's ‘antagonistic pleiotropy' theory and/or Kirkwood's ‘disposable soma' theory for the evolution of senescence [3,30].

First, both cells forage according to how much each has invested into foraging ability, with resource returns on allocating *x* to foraging given by

4.1 $$f(x)=f_{\max}\frac{x}{x+h},$$

where *f*_max_ is the maximal return and *h* is a half-saturation constant (see table 1 for model parameters and their default values). The resources gained from foraging are redistributed among the two cells according to a genetically determined, type-specific reaction norm that allows each cell to signal its ‘need'. If cell 1 signals *s*_1_ and cell 2 *s*_2_, then cell 1 receives *s*_1_/(*s*_1_ + *s*_2_) and cell 2 the rest. This trait potentially allows a degree of altruism between cells to evolve, for if *s*_1_ > *s*_2_, then cell 2 will ‘allow' cell 1 a greater share of the resources than itself, or *vice versa*. The resulting ‘symmetry breaking', i.e. the unequal distribution of resources between cells, might pave the way for the evolution of division of labour. (Note that the ‘cells' in our model could also be thought of as representing distinct ‘castes' in the case of eusocial insect colonies.)

**Table 1.** Model parameters.

parameter	meaning	values in figures 1–3
*N*	population size	10^5^, 10^5^, 10^5^
*f*_max_	maximal foraging returns (resources)	6.0, 6.0, 6.0
*h*	half-saturation constant (resources)	0.3, 0.3, 0.3
*λ*	exponential distribution of damage increments (1/damage)	2.0. 2.0, 2.0
*a*	rate at which repair decreases damage (1/resources)	0.5, 0.5, 0.5
*b*	rate at which reproduction increases with investment (1/resources)	0.1, 0.1, 0.1
*c*	convexity mortality versus damage relation	0.8, 0.8, 0.8
*D*	inverse extrinsic mortality	1000, 1.0, 1.0
*m*_2_	probability of mortality when deleterious allele expressed	0.05, 0.05, 0.05
*r*	probability single cell regenerates adult after death of partner	1.0, (0,1), (0,1)
*μ*	mutation probability reaction norm alleles	0.05, 0.05, 0.05
*σ*	standard deviation Gaussian mutation effect sizes	0.5, 0.5, 0.5
*μ_v_*_0_	mutation probability damage-specific viability loci (neutral to deleterious)	0.001, 0.001, 0.001
*μ_v_*_1_	mutation probability damage-specific viability loci (deleterious to neutral)	0.0001, 0.0001, 0.0001
*μ_d_*	mutation probability damage partition locus	0, 0, 0.05

Second, each cell accumulates damage, by an amount drawn from an exponential distribution with rate parameter *λ*. Each cell then repairs damage according to how much it has allocated to repair. Specifically, given an amount *y* is allocated to repair and an amount of damage *d* before repair, the amount of damage after the repair is given by

4.2 $$d^{\prime }=d\;\exp(-ay),$$

where *a* is a positive constant (table 1). Thus, depending on *y*, damage can increase over the lifetime of the organism (small enough *y*) or decrease (large enough *y*), in line with our first *desideratum*.

Third, each of the two cells produces an offspring with a probability that depends on the amount of resources allocated to the reproduction. Specifically, given an amount of resources *z* allocated to reproduction, the probability of producing a daughter cell is

4.3 p=1−exp(−bz),

where *b* is some positive constant. Thus, if nothing is allocated to reproduction (i.e. *z* = 0), no progeny will be produced (i.e. *p* = 0). Initially, each daughter cell inherits half of the damage of the mother cell. Later, we will relax this assumption and allow for an evolvable, genetically determined reaction norm to dictate the partitioning of damage among the cells. Each daughter cell also inherits resources that are proportional to its mother's investment into reproduction. It then develops into an adult by doubling once, thereby splitting resources and damage equally between itself (henceforth designated type 1) and its daughter cell (designated type 2).

Fourth, three rounds of mortality are applied. In the first round, the probability of cellular death *m*_1_ depends on the amount of cellular damage *d* according to:

4.4 $$m_{1}=(1-c)(d/D)+c(d/D{)}^{4}.$$

This is the same function as used by Ackermann *et al.* [24] in a somewhat similar model of ageing in unicellular organisms. The parameter *c* determines the degree of convexity of the function, from linear (*c* = 0) to strongly convex (*c* = 1), while *D* scales the effect of damage, with smaller values corresponding to high extrinsic mortality and high values to low extrinsic mortality. During the second round of mortality, damage-specific deleterious alleles come in. There are 16 bi-allelic loci with a neutral allele and a deleterious mutant allele. Each locus is expressed in terms of a locus-specific range of damage values (i.e. the first locus for 0 ≤ *d* < *d*_1_, the second locus for *d*_1_ ≤ *d* < *d*_2_ and so on, where the specific boundary values are evenly spaced and cover >99% of the observed damage range). This is intended to mimic a situation where gene expression is conditional on a ‘biomarker' of age—namely damage—which potentially allows the long-term accumulation of deleterious age-specific mutations (Medawar's mutation accumulation theory of ageing [2]). If a deleterious allele is expressed, the probability of mortality is *m*_2_ for each locus. If after the first two rounds of mortality both cells of an individual have died, then the individual also dies (obviously), but if only one of an individual's cells dies, then with probability *r* the surviving cell will double again and regenerate a bicellular adult. During the third round of mortality, the population is culled randomly until *N* adult individuals remain, and a new population cycle of foraging, damage accumulation, repair, reproduction and mortality begins.

The allocation genes for foraging and repair can take any values *x* and *y* on the real line (up to machine precision), which are transformed to proportions *p_x_* and *p_y_* by applying a softmax function, which is defined as

4.5 $$p_{x\;\mathrm{or}\;y}=\frac{\exp(x\;\mathrm{or}\;y)}{1+\exp(x)+\exp(y)}.$$

Allocation to reproduction is then $p_{z}=1-p_{x}-p_{y}$. During the production of daughter cells, the genetic values *x* and *y* mutate with probability *μ*, in which case their value is incremented by drawing from a Gaussian distribution with mean zero and standard deviation *σ*. The 16 bi-allelic loci have biased mutations such that neutral alleles are more likely to mutate to deleterious alleles than *vice versa*.

Below we provide a first, preliminary exploration of this model, especially in light of the potential role of parent–offspring asymmetry and the division of labour for the evolution of ageing. Simulations were run for 10 000 cycles. The code was written in C++, compiled with g++ 9.3.0 and run under Ubuntu 18.04 (code and scripts available on https://github.com/idopen/asymmetry_and_ageing). Data were analysed with R version 3.6.0 [97] in RStudio 1.2.5019 [98]. R packages used were tidyverse [99], cowplot [100] and mgcv [101].

## 5. Results and discussion

### (a) Asymmetry or division of labour are not required for senescence to evolve

**Figure 1.**
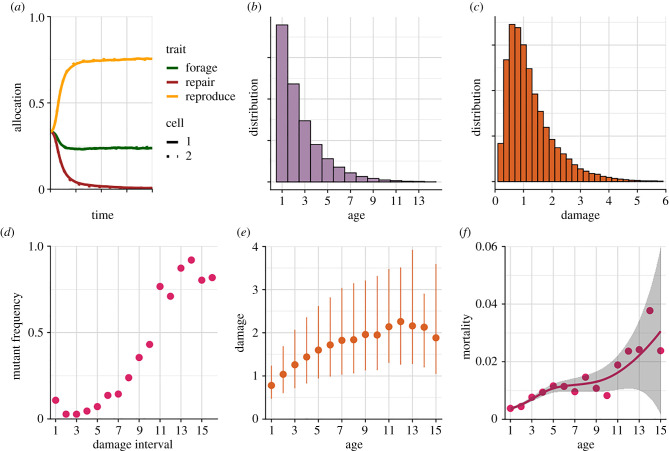
Evolution of senescence in the absence of parent–offspring asymmetry. (*a*) Evolutionary dynamics of cell type-specific allocation traits during the first 1000 cycles. Note that no differentiation between cell types evolves (which makes the dashed lines for cell type 2 hard to distinguish from the solid ones for type 1, owing to their overlap), that repair evolves to zero, and that reproduction and foraging evolve to approximately 0.75 and 0.25, respectively. (*b*) The final age distribution among individuals. (*c*) The final damage distribution among cells. (*d*) Final frequencies of deleterious alleles with damage-specific effects. The 16 intervals are evenly spaced between 0 and 6. Note that deleterious mutations tend to reach higher frequencies when expressed at higher damage levels. (*e*) Accumulation of damage with age (median and 80% intervals). (*f*) Mortality due to deleterious alleles increases with age, indicative of senescence. The curve was fitted with a generalized additive model and the shaded area represents the corresponding 95% confidence band.

Above we have reviewed (mainly verbal) arguments for the proposition that parent–offspring asymmetry (or, more generally, division of labour) is necessary for senescence to evolve, or equivalently, that ageing cannot evolve in the absence of such an asymmetry [23] (also see [3,6,24,29]). As far as we know, however, the logic of this simple proposition has never been tested with a formal model, presumably because the verbal arguments are quite convincing. We were therefore surprised that, contrary to this proposition, our model predicts that ageing can evolve in the absence of any parent–offspring asymmetry (figure 1). Specifically, we assumed that maternal cellular damage is evenly split between mother and daughter cell. Thus, barring mutations, mother and daughter cells are indistinguishable (i.e. symmetrical). To prevent damage accumulation with age from having direct deleterious effects upon survival, which could be regarded as a form of ‘built-in' senescence, we assumed that damage has no direct effect on survival. We did however allow for deleterious mutations with damage-specific effects to mutate into existence from an initial population free of such deleterious alleles and hence not initially subject to any senescence at all. Figure 1*d* shows that the frequencies of such deleterious alleles eventually increase with the amount of damage at which they are expressed. As a result, the accumulation of damage with age (figure 1*e*) leads to an increase of mortality with age (figure 1*f*), i.e. the defining hallmark of senescence.

So why does the above-mentioned verbal argument about parent–offspring asymmetry fail? Here is an instance where a model can help us to sharpen our intuition and to rethink our verbal arguments. We can now see that even if parents and offspring are completely indistinguishable (i.e. symmetrical) with respect to a biomarker of age (in other words, if the biological clock is not being reset in offspring), then it is still the case that individuals (or mother–daughter pairs) with a relatively large amount of damage will be relatively rare and hence that selection will be correspondingly weaker against deleterious alleles that are only expressed in the presence of such an amount of damage. Note that the shape of the frequency distribution of damage (figure 1*c*) is approximately inversely proportional to the distribution of (quasi-)equilibrium frequencies of the corresponding deleterious alleles.

**Figure 2.**
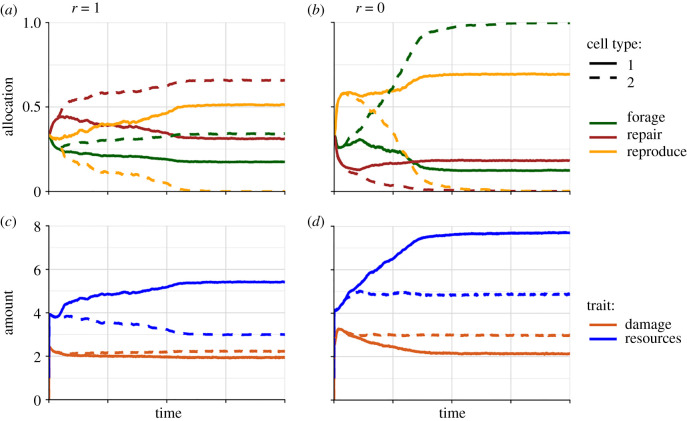
Evolution of division of labour or germ-line–soma separation in the absence of parent–offspring asymmetry. As compared with figure 1, damage now has a direct effect on mortality. (*a*) Evolutionary dynamics of mean cell type-specific allocation traits during 10 000 cycles. Individuals always die when at least one of their two cells dies (*r* = 1). Note that cell type 1 specializes in reproduction (allocation evolves to approximately 0.5), while cell type 2 eventually does not reproduce at all, but allocates more to foraging and repair than cell type 1. (*b*) The same as (*a*) but individuals always survive when one of their cells dies (*r* = 0). Now cell type 2 evolves towards 100% foraging, while allocating nothing to repair and reproduction. (*c,d*) Evolutionary partitioning of resources and damage (multiplied by 10) between cell types. Parameters for (*c*) are identical to those in (*a*), and parameters for (*d*) are identical to those in (*b*). Note that cell type 1, which monopolizes reproduction, ends up with more resources and less damage than cell type 2.

The main argument in favour of parent–offspring symmetry precluding ageing is that, without asymmetry between parent and offspring, the individuals would be indistinguishable so that if they would deteriorate over chronological time and over successive generations, this deterioration would affect all individuals of the lineage simultaneously and equivalently; this could even lead to the disappearance of the whole lineage [5,23,24,35]. However, we do not find the logic of this argument compelling. First, even if within generations cellular damage tends to increase, damage may decrease between generations when damage is distributed equally (or even slightly unequally) between mother and daughter cells. Indeed, even with equal partitioning of damage, cell division may be seen as ‘rejuvenating' for the maternal cell since she will have less cellular damage after division than before. Thus, the amount of damage may reach an equilibrium where it no longer increases from one generation to the next (e.g. as in figure 2*c,d*), making lineage extinction far from certain. Second, while it is true that with perfect symmetry one cannot differentiate between the biological age of the parent and that of the offspring, this does not imply that parent and offspring do not age: they might simply age at the same rate but they might undergo senescence nonetheless. In such a scenario, different parent–offspring lineages might age at different rates, for example, when they accumulate and/or repair damage at different rates. Interestingly, even though Partridge & Barton [23, p. 310] argue that parent–offspring asymmetry is the central criterion for the evolution of ageing, they also make a hypothetical case in favour of the compatibility of symmetry and ageing: ‘Consider a multicellular organism in which randomly chosen cells dedifferentiate and divide to produce germ cells until the organism has completely turned into gametes, and where there is therefore no distinction between soma and germ line. Will this creature, as an individual, age? As gamete-production proceeds, it will have a non-zero probability of death or of becoming unable to produce gametes. The intensity of selection on survival and rate of gamete-production will therefore decline with age and ageing will evolve'. In this scenario, there is no separation (i.e. asymmetry, division of labour) between germ-line and soma, yet ageing can still evolve. Similarly, and as supported by our simulations, even if perfect parent–offspring symmetry were to exist (e.g. in some hypothetical unicellular organism), the individual cells and the cell lineages created at cell division could still undergo ageing. We therefore suggest that parent–offspring asymmetry (or, more generally, division of labour) might not be a necessary condition for the evolution of ageing. If, however, asymmetry or division of labour does evolve, the constituent ‘parts' of the system (e.g. the cellular lineages produced by bacteria at cell division, the different cell types of a multicellular organism or the different castes in a eusocial insect colony) are expected to age at different rates (or at the same rate but with different intercept).

From this perspective, and as argued by Hamilton [4], the evolution of senescence might therefore be inevitable, and all organisms (and/or their constituent ‘parts') might be expected to age but they might do so at different rates and/or with different intercepts in terms of their age-dependent mortality trajectories. Cases of slow ageing might be explained by the notion that the force of selection against higher mortality in a given age class is proportional to the product of two factors: the relative frequency of the age class and the reproductive value (i.e. fitness) of the members of that age class. The rarity of an age class can thus be offset by its reproductive value, and so slow ageing or even the opposite of ageing (i.e. an age-progressive increase of fitness) might occur (see e.g. [58]).

### (b) Asymmetric resource partitioning favours germ-line–soma separation

In the example shown in figure 1, there is clearly no evolutionary divergence between the two cell types; both types make the same proportional allocation of their resources to foraging, damage repair and reproduction during the entire evolutionary trajectory. It turns out, however, that specialization (division of labour) can evolve quite easily in our model. Instead of presenting a full analysis of our model (which is beyond the scope of this paper), we present some proofs of principle and offer some tentative conjectures.

Figure 2 shows two examples of the evolution of specialization or germ-line–soma separation. In both cases (*a* and *c* versus *b* and *d*), cell type 1 eventually comes to completely monopolize reproduction while cell type 2 invests more in foraging. The main difference from figure 1 in terms of parameter values is that for figure 2 we allowed a direct, strongly deleterious effect of cellular damage upon cellular survival (by decreasing the value of parameter *D* from 1000 to 1.0; table 1). The most extreme case of divergence can be seen in figure 2*b*, where we assumed that a bicellular individual can be regenerated after one of its two cells has died. Here one cell type allocates 100% of the resources to foraging. Since this type repairs no damage, it will accumulate more damage (figure 2*d*) and die at a higher rate than its reproductive partner (i.e. the ‘germ-line'), but since it will then become replaced by a new daughter cell of its partner, it functions literally as the ‘disposable soma' [30–32]. Thus, not only do we observe functional specialization of cells but we also observe that the cells age at different rates, even if their fates are intimately linked (in figure 2*a*,*c* the death of one of the two cells always implies the death of the other, i.e. *r* = 1). Although we have phrased our model in terms of two cells composing the same body, we might also have phrased it in terms of two ‘castes' of a social species. It is well known that in social insects, which are characterized by division of labour between castes, rates of ageing can differ dramatically between castes [68–72].

What is it that makes germ-line–soma separation evolve quite readily in our model? We did not have to search long and hard to find ‘suitable' parameter combinations—on the contrary. We also did not choose functions (equations (4.1)–(4.4)) that bias the results in favour of functional specialization of cells. Indeed, all our assumed functions are concave or decelerating, meaning that fitness benefits show diminishing returns with respect to the causal predictor variables (allocation and damage). It is well known that convex or accelerating functions tend to favour specialization [49]; hence, if anything, our choice of functions biased the results *against* specialization. Instead, we conjecture that the crucial factor for the evolution of specialization in our model is the assumed ability of the two cells to divide resources unequally among themselves, which can be regarded as a form of altruism by cells that ‘accept' a smaller share. In our examples (figure 2*c,d*), it is always the non-reproducing cell (the ‘soma') that ends up conceding a larger share of the resources to the reproducing cell (the ‘germ-line'). The logic is that if two cells have different amounts of resources available, even if the two cells must obey the exact same set of trade-offs (as defined by equations (4.1)–(4.4)), they will almost certainly have different fitness-maximizing allocation strategies, which paves the way for selection to favour the division of labour. To test this line of reasoning, we re-ran our simulations without giving cells the ability to evolve unequal partitioning of resources, and indeed specialization no longer evolved in this case (results not shown). In addition to this role of resource partitioning, we also found in simulation experiments that higher extrinsic mortality (i.e. lower *D* values) tends to favour specialization (figure 1 versus figure 2). We hypothesize that the resulting weaker relation between damage and mortality (equation (4.4)) makes overall fitness less sensitive to variation in resource availability between the two cells, making it harder for selection to ‘see' differences in allocation strategies between cells. Given the potentially important role for altruistic behaviour that we see as driving the evolution of division of labour, it might be interesting to develop a version of our model where the two cooperating cells are somewhat less closely related. A standard kin selection argument predicts that a division of labour would evolve less readily in that case.

Which of the cell types becomes the ‘germ-line' seems to be random in our model (out of 10 runs, each type became the germ-line five times). Interestingly, it is easy to break this symmetry by allowing for an asymmetry in mutation rates between cell types. If during development, the daughter cell (type 2) of the initial unicellular individual (type 1) undergoes an extra round of mutation, then the germ-line invariably evolves in cell type 1, thus having a slightly smaller mutational load. This is reminiscent of the observations by Aanen [53], who noted that, under certain conditions, the mother cells tend to keep the ‘original' template DNA, while the daughter cells are more likely to receive the slightly more damaged copy of the template.

**Figure 3.**
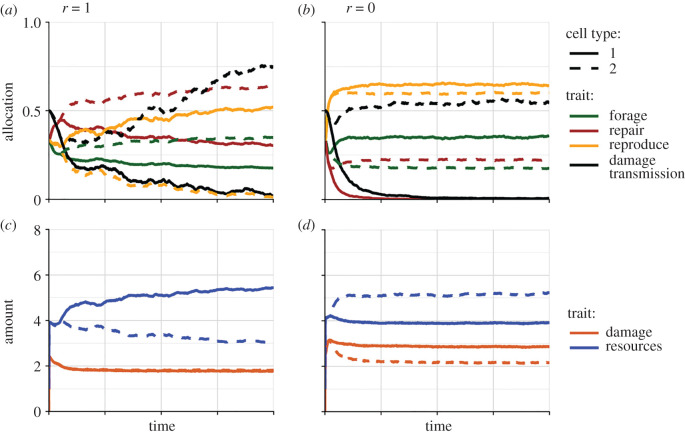
Evolution of cellular specialization when partitioning of damage (black lines) can evolve; otherwise, model parameters are exactly the same as in figure 2. In *a* and *c* (where *r* = 1), one cell type again comes to monopolize reproduction (*a*, orange lines), while the other invests more into foraging (*a*, green lines) and concedes more resources to the reproductive cell (*c*, blue lines). The reproductive cells confine all damage to themselves, producing damage-free (fully ‘rejuvenated') daughter cells. The cells appear to age at the same rates as they accumulate the same amount of damage (*c*, orange lines). In (*b*) and (*d*) (*r* = 0), reproductive monopolization does not evolve, but one cell type confines all cellular damage to itself when reproducing, while the other passes on a little over half the damage.

We took this asymmetric inheritance of damage a step further, following the model of Ackermann *et al*. [24], by studying a version of our model where the partitioning of damage is an evolvable trait (figure 3), with equal partitioning being the ancestral state. So far, we have assumed that cellular damage is split equally between mother and daughter cells during cell division; indeed, all else being equal, this is probably the thermodynamically favoured outcome which maximizes cellular entropy. Our extended model is more general than the one by Ackermann *et al.* [24] in that we also allow damage partitioning to differ between cell types. Not surprisingly, this additional degree of freedom enriches the range of possible model outcomes. Typically, we observed that one cell type confines all damage to itself while producing damage-free daughter cells, while the other cell type passes on roughly half of the damage to its offspring. However, while in previous simulations we tended to observe either no specialization (figure 1) or the evolution of reproductive monopolization by one cell type (figure 2), we now observed both reproductive monopolization (figure 3*a*,*c*) and cases where both cell types reproduce at similar rates but nevertheless have very different damage retention rates (figure 3*b*,*d*).

## 6. Conclusion

The key findings from our simulation model, i.e. our tentative conjectures, can be briefly summarized as follows:

(1) Parent–offspring asymmetry (or, more generally, division of labour) is not a necessary condition for the evolutionary origin of ageing, contrary to previous claims. For example, even if the damage is partitioned equally between a mother and a daughter cell, both cells can still undergo senescence.
(2) If asymmetry (or division of labour) evolves, the different ‘constituent parts' of an organismal ‘system' can exhibit different rates of ageing, with one part (e.g. the germ-line) ageing more slowly than the other part (e.g. the soma), in line with ‘trade-off' theories for the evolution of ageing, e.g. Williams' ‘antagonistic pleiotropy' theory and/or Kirkwood's ‘disposable soma' theory.
(3) Asymmetric resource partitioning among ‘parts’ strongly promotes the evolution of germ-line–soma separation in our model. This sort of division of labour presumably requires altruistic cooperation among the ‘parts', which might be facilitated by a high degree of genetic relatedness among them.
(4) Selection might favour cell type-specific partitioning of damage between cell types, with one cell type specializing in reproduction and retaining the bulk of the damage, thus producing ‘rejuvenated', damage-free daughter cells, while the non-reproducing cell type distributes damage more or less equally.
(5) Thus, although the evolutionary origin of ageing seems to require no particular asymmetry other than an age-dependent asymmetry in the force of selection (high early in life, low late in life) operating in populations with asymmetric (i.e. non-uniform) age-dependent distributions of ‘biomarkers' of senescence, small initial asymmetries in resource availability and trade-offs between the ‘parts' of an organismal ‘system' may trigger the evolution of large asymmetries in the form of division of labour, germ-line–soma separation and variable rates of ageing between the system's parts.

## Data accessibility

This article has no additional data.

## References

[1] Medawar PB. 1948 Old age and natural death. Modern Q. **2**, 30-49.

[2] Medawar PB. 1952 An unsolved problem of biology. London, UK: H.K. Lewis.

[3] Williams GC. 1957 Pleiotropy, natural selection, and the evolution of senescence. Evolution **11**, 398-411. (10.1111/j.1558-5646.1957.tb02911.x)

[4] Hamilton WD. 1966 Moulding of senescence by natural selection*.* J. Theor. Biol. **12**, 12-45. (10.1016/0022-5193(66)90184-6) 6015424

[5] Rose MR. 1991 Evolutionary biology of aging. Oxford, UK: Oxford University Press.

[6] Charlesworth B. 1980 Evolution in age-structured populations, 1st edn. Cambridge, UK: Cambridge University Press.

[7] Charlesworth B. 1994 Evolution in age-structured populations, 2nd edn. Cambridge, UK: Cambridge University Press.

[8] Flatt T, Partridge L. 2018 Horizons in the evolution of aging. BMC Biol. **16**, 93. (10.1186/s12915-018-0562-z) 30124168 PMC6100731

[9] Finch CE. 1990 Longevity, senescence, and the genome. Chicago, IL: University of Chicago Press.

[10] Jones OR et al. 2014 Diversity of ageing across the tree of life. Nature **505**, 169-173. (10.1038/nature12789) 24317695 PMC4157354

[11] Shefferson RP, Jones OR, Salguero-Gómez R. 2017 The evolution of senescence in the tree of life. Cambridge, UK: Cambridge University Press.

[12] Vaupel JW, Baudisch A, Dolling M, Roach D, Gampe J. 2004 The case for negative senescence. Theor. Pop. Biol. **65**, 339-351. (10.1016/j.tpb.2003.12.003) 15136009

[13] Baudisch A. 2008 Inevitable aging? Berlin, Germany: Springer.

[14] Schaible R, Scheuerlein A, Dańko MJ, Gampe J, Martínez DE, Vaupel JW. 2015 Constant mortality and fertility over age in *Hydra*. Proc. Natl Acad. Sci. USA **112**, 15 701-15 706. (10.1073/pnas.1521002112) PMC4697432 26644561

[15] Ruby JG, Smith M, Buffenstein R. 2018 Naked mole-rat mortality rates defy Gompertzian laws by not increasing with age. eLife **7**, e31157. (10.7554/eLife.31157) 29364116 PMC5783610

[16] Munné-Bosch S. 2015 Senescence: is it universal or not? Trends Plant Sci. **20**, 713-720. (10.1016/j.tplants.2015.07.009) 26442681

[17] Stearns SC. 1992 The evolution of life histories. Oxford, UK: Oxford University Press.

[18] Stearns SC. 2005 Issues in evolutionary medicine. Am. J. Hum. Biol. **17**, 131-140. (10.1002/ajhb.20105) 15736177

[19] Roper M, Capdevila P, Salguero-Gómez R. 2019 Senescence: still an unsolved problem of biology. *bioRxiv*, 739730. (10.1101/739730) PMC8292751 34284628

[20] Weismann A. 1882 Über die Dauer des Lebens *[*On longevity*]*. *[In German.]*. Jena, Germany: Gustav Fischer.

[21] Weismann A. 1891 Essays upon heredity and kindred biological problems, 2nd edn. Oxford, UK: Clarendon Press.

[22] Weismann A. 1892 Die Kontinuität des Keimplasmas als Grundlage einer Theorie der Vererbung *[*The germ-plasm: a theory of heredity*]*. *[In German.]*. Jena, Germany: Gustav Fischer.

[23] Partridge L, Barton NH. 1993 Optimality, mutation and the evolution of ageing. Nature **362**, 305-311. (10.1038/362305a0) 8455716

[24] Ackermann M, Chao L, Bergstrom CT, Doebeli M. 2007 On the evolutionary origin of aging. Aging Cell **6**, 235-244. (10.1111/j.1474-9726.2007.00281.x) 17376147 PMC2049046

[25] Kirkwood TBL. 2005 Asymmetry and the origins of ageing. Mech. Ageing Dev. **126**, 533-534. (10.1016/j.mad.2005.02.001) 15811422

[26] Martínez DE, Levinton JS. 1992 Asexual metazoans undergo senescence. Proc. Natl Acad. Sci. USA **89**, 9920-9923. (10.1073/pnas.89.20.9920) 11607334 PMC50245

[27] Goldsby HJ, Knoester DB, Ofria C, Kerr B. 2014 The evolutionary origin of somatic cells under the dirty work hypothesis. PLoS Biol. **12**, e1001858. (10.1371/journal.pbio.1001858) 24823361 PMC4019463

[28] Kirkwood TBL, Cremer T. 1982 Cytogerontology since 1881: a reappraisal of August Weismann and a review of modern progress. Hum. Genet. **60**, 101-121. (10.1007/bf00569695) 7042533

[29] Bell G. 1984 Evolutionary and nonevolutionary theories of senescence. Am. Nat. **124**, 600-603. (10.1086/284300)

[30] Kirkwood TBL. 1977 Evolution of ageing. Nature **270**, 301-304. (10.1038/270301a0) 593350

[31] Kirkwood TBL. 1987 Immortality of the germ-line versus disposability of the soma. In Evolution of longevity in animals: a comparative approach (eds AD Woodhead, KH Thompson), pp. 209-218. Boston, MA: Springer. 10.1007/978-1-4613-1939-9_15 3435387

[32] Kirkwood T, Holliday R. 1979 The evolution of ageing and longevity. Proc. R. Soc. Lond. B **205**, 531-546. (10.1098/rspb.1979.0083) 42059

[33] Bell G. 1988 Sex and death in Protoza. The history of an obsession. Cambridge, UK: Cambridge University Press.

[34] Nyström T. 2003 Conditional senescence in bacteria: death of the immortals. Mol. Microbiol. **48**, 17-23. (10.1046/j.1365-2958.2003.03385.x) 12657042

[35] Kirkwood TBL, Austad SN. 2000 Why do we age? Nature **408**, 233-238. (10.1038/35041682) 11089980

[36] Ackermann M, Stearns SC, Jenal U. 2003 Senescence in a bacterium with asymmetric division. Science **300**, 1920-1920. (10.1126/science.1083532) 12817142

[37] Stewart EJ, Madden R, Paul G, Taddei F. 2005 Aging and death in an organism that reproduces by morphologically symmetric division. PLoS Biol. **3**, e45. (10.1371/journal.pbio.0030045) 15685293 PMC546039

[38] Lindner AB, Madden R, Demarez A, Stewart EJ, Taddei F. 2008 Asymmetric segregation of protein aggregates is associated with cellular aging and rejuvenation. Proc. Natl Acad. Sci. USA **105**, 3076-3081. (10.1073/pnas.0708931105) 18287048 PMC2268587

[39] Proenca AM, Rang CU, Qiu A, Shi C, Chao L. 2019 Cell aging preserves cellular immortality in the presence of lethal levels of damage. PLoS Biol. **17**, e3000266. (10.1371/journal.pbio.3000266) 31120870 PMC6532838

[40] Jazwinski SM. 1993 The genetics of aging in the yeast *Saccharomyces cerevisiae*. Genetica **91**, 35-51. (10.1007/BF01435986) 8125278

[41] Lai CY, Jaruga E, Borghouts C, Jazwinski SM. 2002 A mutation in the ATP2 gene abrogates the age asymmetry between mother and daughter cells of the yeast *Saccharomyces cerevisiae*. Genetics **162**, 73-87. 12242224 10.1093/genetics/162.1.73 PMC1462265

[42] Kirkwood TBL. 2005 Understanding the odd science of aging. Cell **120**, 437-447. (10.1016/j.cell.2005.01.027) 15734677

[43] Watve M, Parab S, Jogdand P, Keni S. 2006 Aging may be a conditional strategic choice and not an inevitable outcome for bacteria. Proc. Natl Acad. Sci. USA **103**, 14 831-14 835. (10.1073/pnas.0606499103) PMC1595437 17001004

[44] Evans SN, Steinsaltz D. 2007 Damage segregation at fissioning may increase growth rates: a superprocess model. Theor. Pop. Biol. **71**, 473-490. (10.1016/j.tpb.2007.02.004) 17442356 PMC2430589

[45] Erjavec N, Cvijovic M, Klipp E, Nyström T. 2008 Selective benefits of damage partitioning in unicellular systems and its effects on aging. Proc. Natl Acad. Sci. USA **105**, 18 764-18 769. (10.1073/pnas.0804550105) PMC2596250 19020097

[46] Johnson LR, Mangel M. 2006 Life histories and the evolution of aging in bacteria and other single-celled organisms. Mech. Ageing Dev. **127**, 786-793. (10.1016/j.mad.2006.07.004) 16899276

[47] Szathmáry E, Maynard SJ. 1995 The major evolutionary transitions. Nature **374**, 227-232. (10.1038/374227a0) 7885442

[48] Maynard Smith J, Szathmáry E. 1995 The major transitions in evolution. Oxford, UK: Oxford University Press.

[49] Rueffler C, Hermisson J, Wagner GP. 2012 Evolution of functional specialization and division of labor. Proc. Natl Acad. Sci. USA **109**, 1830-1831. (10.1073/pnas.1110521109) PMC3277576 22308336

[50] Nyström T. 2011 Spatial protein quality control and the evolution of lineage-specific ageing. Phil. Trans. R. Soc. Lond. B **366**, 71-75. (10.1098/rstb.2010.0282) 21115532 PMC3001311

[51] Buss LW. 1987 The evolution of individuality. Princeton, NJ: Princeton University Press.

[52] Jones DL. 2007 Aging and the germ line: where mortality and immortality meet. Stem Cell Rev. **3**, 192-200. (10.1007/s12015-007-0009-3) 17917132

[53] Aanen DK. 2018 Social immunity: the disposable individual. Curr. Biol. **28**, R322-R324. (10.1016/j.cub.2018.02.050) 29614293

[54] Keller L, Genoud M. 1997 Extraordinary lifespans in ants: a test of evolutionary theories of ageing. Nature **389**, 958-960. (10.1038/40130)

[55] Boomsma JJ, Huszár DB, Pedersen JS. 2014 The evolution of multiqueen breeding in eusocial lineages with permanent physically differentiated castes. Anim. Behav. **92**, 241-252. (10.1016/j.anbehav.2014.03.005)

[56] Boomsma JJ, Gawne R. 2018 Superorganismality and caste differentiation as points of no return: how the major evolutionary transitions were lost in translation. Biol. Rev. **93**, 28-54. (10.1111/brv.12330) 28508537

[57] Kramer BH, Schaible R. 2013 Life span evolution in eusocial workers—a theoretical approach to understanding the effects of extrinsic mortality in a hierarchical system. PLoS ONE **8**, e61813. (10.1371/journal.pone.0061813) 23596527 PMC3626611

[58] Kramer BH, van Doorn GS, Weissing FJ, Pen I. 2016 Lifespan divergence between social insect castes: challenges and opportunities for evolutionary theories of aging. Curr. Opin. Insect Sci. **16**, 76-80. (10.1016/j.cois.2016.05.012) 27720054

[59] Chapuisat M, Keller L. 2002 Division of labour influences the rate of ageing in weaver ant workers. Proc. R. Soc. Lond. B **269**, 909-913. (10.1098/rspb.2002.1962) PMC1690981 12028773

[60] Maynard Smith J. 1962 Review lectures on senescence. I. The causes of ageing. Proc. R. Soc. Lond. B **157**, 115-127. (10.1098/rspb.1962.0065) 14042285

[61] Cevenini E, Invidia L, Lescai F, Salvioli S, Tieri P, Castellani G, Franceschi C. 2008 Human models of aging and longevity. Exp. Opin. Biol. Therap. **8**, 1393-1405. (10.1517/14712598.8.9.1393) 18694357

[62] Walker LC, Herndon JG. 2010 Mosaic aging. Med. Hypoth. **74**, 1048-1051. (10.1016/j.mehy.2009.12.031) PMC2854836 20110150

[63] Herndon LA et al. 2002 Stochastic and genetic factors influence tissue-specific decline in ageing *C. elegans*. Nature **419**, 808-814. (10.1038/nature01135) 12397350

[64] Tuttle CSL, Waaijer MEC, Slee-Valentijn MS, Stijnen T, Westendorp R, Maier AB. 2020 Cellular senescence and chronological age in various human tissues: a systematic review and meta-analysis. Aging Cell **19**, e13083. (10.1111/acel.13083) 31808308 PMC6996941

[65] Schaum N et al*.* 2020 Ageing hallmarks exhibit organ-specific temporal signatures. Nature **583**, 596-602. (10.1038/s41586-020-2499-y) 32669715 PMC7757734

[66] Monaghan P, Metcalfe NB. 2019 The deteriorating soma and the indispensable germline: gamete senescence and offspring fitness. Proc. R. Soc. B **286**, 20192187. (10.1098/rspb.2019.2187) PMC6939927 31847776

[67] Li Y et al. 2020 A programmable fate decision landscape underlies single-cell aging in yeast. Science **369**, 325-329. (10.1126/science.aax9552) 32675375 PMC7437498

[68] Bourke AFG. 1999 Colony size, social complexity and reproductive conflict in social insects. J. Evol. Biol. **12**, 245-257. (10.1046/j.1420-9101.1999.00028.x)

[69] Kramer BH, Schaible R. 2013 Colony size explains the lifespan differences between queens and workers in eusocial Hymenoptera. Biol. J. Linn. Soc. **109**, 710-724. (10.1111/bij.12072)

[70] Toth AL, Sumner S, Jeanne RL. 2016 Patterns of longevity across a sociality gradient in vespid wasps. Curr. Opin. Insect Sci. **16**, 28-35. (10.1016/j.cois.2016.05.006) 27720047

[71] Korb J, Thorne B. 2017 Sociality in termites. In Comparative social evolution (eds DR Rubenstein, P Abbot), pp. 124-153. Cambridge, UK: Cambridge University Press.

[72] Monroy KJM, Meusemann K, Korb J. 2019 Long live the queen, the king and the commoner? Transcript expression differences between old and young in the termite *Cryptotermes secundus*. PLoS ONE **14**, e0210371. (10.1371/journal.pone.0210371) 30759161 PMC6373952

[73] Bernard C, Compagnoni A, Salguero-Gómez R. 2020 Testing Finch's hypothesis: the role of organismal modularity on the escape from actuarial senescence. Funct. Ecol. **34**, 88-106. (10.1111/1365-2435.13486)

[74] Bell G. 1985 The origin and early evolution of germ cells as illustrated by the Volvocales. In The origin and evolution of sex (eds HO Halvorson, A Mornoy), pp. 221-256. New York, NY: Alan R. Liss.

[75] Kirk DL. 1988 The ontogeny and phylogeny of cellular differentiation in *Volvox*. Trends Genet. **4**, 32-36. (10.1016/0168-9525(88)90063-7) 3072717

[76] Koufopanou V. 1994 The evolution of soma in the Volvocales. Am. Nat. **143**, 907-931. (10.1086/285639)

[77] Michod RE. 2006 The group covariance effect and fitness trade-offs during evolutionary transitions in individuality. Proc. Natl Acad. Sci. USA **103**, 9113. (10.1073/pnas.0601080103) 16751277 PMC1482575

[78] Michod RE. 2007 Evolution of individuality during the transition from unicellular to multicellular life. Proc. Natl Acad. Sci. USA **104**, 8613-8618. (10.1073/pnas.0701489104) 17494748 PMC1876437

[79] Nedelcu A, Michod RE. 2011 Molecular mechanisms of life history trade-offs and the evolution of multicellular complexity in volvocalean green algae. In Mechanisms of life history evolution: the genetics and physiology of life history traits and trade-offs (eds T Flatt, A Heyland), pp. 271-283. Oxford, UK: Oxford University Press.

[80] Wagner GP. 2018 Homology, genes, and evolutionary innovation. Princeton, NJ: Princeton University Press.

[81] Gavrilets S. 2010 Rapid transition towards the division of labor via evolution of developmental plasticity. PLoS Comp. Biol. **6**, e1000805. (10.1371/pcbi.1000805.t001) PMC2883585 20548941

[82] Michod RE, Viossat Y, Solari CA, Hurand M, Nedelcu AM. 2006 Life-history evolution and the origin of multicellularity. J. Theor. Biol. **239**, 257-272. (10.1016/j.jtbi.2005.08.043) 16288782

[83] Nelson P, Masel J. 2017 Intercellular competition and the inevitability of multicellular aging. Proc. Natl Acad. Sci. USA **114**, 12 982-12 987. (10.1073/pnas.1618854114) PMC5724245 29087299

[84] Blagosklonny MV. 2012 Answering the ultimate question “What is the proximal cause of aging?”. Aging (Albany, NY) **4**, 861-877. (10.18632/aging.100525) 23425777 PMC3615154

[85] Baudisch A, Salguero-Gómez R, Jones OR, Wrycza T, Mbeau-Ache C, Franco M, Colchero, F. 2013 The pace and shape of senescence in angiosperms. J. Ecol. **101**, 596-606. (10.1111/1365-2745.12084)

[86] Petralia RS, Mattson MP, Yao PJ. 2014 Aging and longevity in the simplest animals and the quest for immortality. Ageing Res. Rev. **16**, 66-82. (10.1016/j.arr.2014.05.003) 24910306 PMC4133289

[87] Rando TA. 2006 Stem cells, ageing and the quest for immortality. Nature **441**, 1080-1086. (10.1038/nature04958) 16810243

[88] Dijkwel PP, Lai AG. 2019 Hypothesis: plant stem cells hold the key to extreme longevity. Transl. Med. Aging **3**, 14-16. (10.1016/j.tma.2018.12.002).

[89] Aanen DK, Debets AJM. 2019 Mutation-rate plasticity and the germline of unicellular organisms. Proc. R. Soc. B **286**, 20190128. (10.1098/rspb.2019.0128) PMC6532511 31039713

[90] Cairns J. 1975 Mutation selection and the natural history of cancer. Nature **255****,** 197-200. (10.1038/255197a0) 1143315

[91] Aanen DK. 2014 How a long-lived fungus keeps mutations in check. Science **346**, 922-923. (10.1126/science.1261401) 25414293

[92] Aanen DK. 2019 Germline evolution: sequestered cells or immortal strands? Curr. Biol. **29**, R799-R801. (10.1016/j.cub.2019.07.033) 31430477

[93] Rosenberger R, Kessel M. 1968 Nonrandom sister chromatid segregation and nuclear migration in hyphae of *Aspergillus nidulans**.* J. Bacteriol. **96**, 1208-1213. (10.1128/JB.96.4.1208-1213.1968) 5685997 PMC252436

[94] Hiltunen M, Grudzinska-Sterno M, Wallerman O, Ryberg M, Johannesson H. 2019 Maintenance of high genome integrity over vegetative growth in the fairy-ring mushroom *Marasmius oreades*. Curr. Biol. **29**, 2758-2765. (10.1016/j.cub.2019.07.025) 31402298

[95] Ispolatov I, Ackermann M, Doebeli M. 2012 Division of labour and the evolution of multicellularity. Proc. R. Soc. B **279**, 1768-1776. (10.1098/rspb.2011.1999) PMC3297448 22158952

[96] Radzvilavicius AL, Hadjivasiliou Z, Pomiankowski A, Lane, N. 2016 Selection for mitochondrial quality drives evolution of the germline. PLoS Biol. **14**, e2000410. (10.1371/journal.pbio.2000410) 27997535 PMC5172535

[97] R Core Team. 2020 R: a language and environment for statistical computing. Vienna, Austria: R Foundation for Statistical Computing. See https://www.R-project.org/.

[98] RStudio Team. 2019 RStudio: integrated development for R. Boston, MA: RStudio, Inc. See http://www.rstudio.com.

[99] Wickham H et al. 2019 Welcome to the tidyverse. J. Open Source Softw. **4**, 1686. (10.21105/joss.01686)

[100] Wilke CO. 2019 *cowplot: Streamlined plot theme and plot annotations for ‘ggplot2’. R package version 1.0.0*. See https://CRAN.R-project.org/package=cowplot.

[101] Wood SN. 2011 Fast stable restricted maximum likelihood and marginal likelihood estimation of semiparametric generalized linear models. J. R. Stat. Soc. B **73**, 3-36. (10.1111/j.1467-9868.2010.00749.x)

